# Prevalence of Childhood Obesity in Indian Expatriate Children in Dubai: A Cross-Sectional Analytical Study

**DOI:** 10.7759/cureus.63202

**Published:** 2024-06-26

**Authors:** Spriha Pandey, Arvind Kavishwar, Maneesha Pandey

**Affiliations:** 1 Basic Sciences, Gems Modern Academy, Dubai, ARE; 2 Non Communicable Diseases, Indian Council of Medical Research National Institute for Research in Tribal Health (ICMR-NIRTH), Jabalpur, IND; 3 Endocrinology, Diabetes and Metabolism, Aster Jubilee Medical Center, Dubai, ARE

**Keywords:** cross-sectional analytical study, overweight, obesity, prevalence, school children

## Abstract

Background

Childhood obesity is one of the most prevalent nutritional disorders affecting children across the world, which further leads to diabetes, hypertension, coronary artery disease, and fatty liver disease in adulthood. The magnitude of this problem among Indian expatriates in the United Arab Emirates (UAE) has not been investigated before. This study delves into the prevalence of childhood obesity among this demographic and also provides a comparative analysis of the prevalence of obesity in UAE citizens and children in India.

Methodology

This is a cross-sectional study that investigates the prevalence of obesity in 3,698 students of a single Indian school in the UAE. Anonymous anthropometric data of these children of age range four to 18 years were analyzed. The International Obesity Task Force (IOTF), World Health Organization (WHO), and Centers for Disease Control (CDC) reference methods were used to calculate the prevalence of overweight, obesity, and extreme obesity.

Results

According to CDC guidelines, the prevalence of body mass index (BMI) ≥ 85th percentile, ≥ 95th percentile, and ≥ 99th percentile stands at 32.74%, 13.68%, and 5.1%, respectively. Children particularly boys aged more than 10 years are at a higher risk of being overweight, obese, and extremely obese (p = < 0.05). In children aged 10 years or less, as the age increases, they tend to have a higher BMI percentile and this is particularly prominent in boys (Pearson correlation coefficient 0.227). Conversely, in those over the age of 10 years, the BMI percentile decreases with age, particularly noticeable in girls, albeit without statistical significance.

Conclusion

Approximately one-third of school-aged Indian expatriates in the UAE are overweight, obese, or extremely obese. Our study, when contrasted with earlier studies, reveals that Emirati teenagers exhibit a higher prevalence of overweight and obesity compared to their Indian counterparts in the UAE. Similarly, the prevalence of childhood obesity among Indian expatriates in the UAE surpasses that among children residing in India.

## Introduction

Obesity is a chronic complex disease defined by excessive fat deposits that can impair health [1]. It is caused as a result of abnormal growth of adipose tissue either due to the enlargement of fat cell size (hypertrophic obesity), an increase in the number of fat cells (hyperplastic obesity), or a combination of both [2]. The obesity epidemic has been spreading rampantly over the last few decades due to the consumption of processed food packed with excessive sugar as well as inadequate physical activity, making childhood obesity one of the most serious problems affecting children across the world today. Over 390 million children and adolescents worldwide, aged five to 19 years were overweight in 2022 [1]. It is estimated that 41 million children under the age of five years were living with overweight or obesity in 2016 [3]. The rate of overweight (including obesity) among children and teenagers aged five to 19 has seen a significant increase, soaring from merely 8% in 1990 to 20% in 2022. This rise has been consistent in both girls and boys, with 19% of girls and 21% of boys being classified as overweight in 2022 [1]. It has been estimated that each year an additional 0.5 to 1% of children gain excess body fat, and up to 32% of these individuals become overweight and 8% become obese [4]. Obesity has many preposterous implications for the youth of today. In the long term, childhood obesity can lead to hypertension, dyslipidemia, type 2 diabetes, coronary heart disease, stroke, gallbladder disease, osteoarthritis, sleep apnea, and breathing problems, it is even linked with mental illnesses such as clinical depression, anxiety, body dysmorphia disorder etcetera [5]. A few studies [6,7] have reported that childhood obesity is associated with a significantly increased risk for cardiovascular events in adult life, even if body weight had meanwhile normalized.

According to the 2022 statistics from the periodic examination of school students, the obesity rate among children and adolescents aged five to 17 years has reached 17.35% in the United Arab Emirates (UAE) [8]. The rate of childhood obesity was increasing apace in the UAE even before the pandemic changed everything: from 12% of children in 2018 to 17.4% in 2020. The largest childhood obesity study conducted in the UAE was conducted in 2015, taking data from over 44000 children across the UAE [9]. This study largely considered children who were citizens of the UAE. Similarly, though there are many studies [10-12] that investigated the burden of obesity and associated risk factors in the school children of UAE providing insightful information, these studies have missed the cause of childhood obesity in Indian expatriate children living in the UAE. About 37.96 % of the UAE population is Indian, with more than 3.86 million Indians living in the country [13]. Thus research on the prevalence and causes of obesity in Indian expatriates living in the UAE is pivotal to control this ever-growing epidemic. Along with this, a relative comparison of the prevalence of childhood obesity in Indian expatriates to that in UAE citizens and children living in India is also essential for accurately attributing childhood obesity to environmental and genetic factors.

An understanding and study of the trends of obesity provides us with an efficient mechanism to control this epidemic. A greater understanding of obesity across different nationalities and ethnicities as well as immigrants living in other countries helps us identify the causes of obesity in children and develop methods to control it. This study explores the prevalence and trends of childhood obesity in Indian expatriates living in the UAE and provides a comparative analysis of the prevalence of obesity in UAE citizens and children living in India. To the best of our knowledge, this is the first such study in Indian expatriates living in the UAE.

## Materials and methods

Approval of the Institutional Ethics Committee was taken before the study vide memo no 25 dated December 18, 2023. This was a cross-sectional analytical study done in Dubai, United Arab Emirates. After obtaining permission from the school administration, the age, gender, and anthropometric measurements of 3,719 students of a single school, Gems Modern Academy, were retrospectively assessed. The data of 21 students was excluded from the study as these were differently abled children, children suffering from mental health conditions, and children with chronic illnesses like Diabetes and thyroid disorders. The data utilized in this study consists of age, gender, height, and weight measurements of the remaining 3,698 students of the school taken in the annual health checkup performed in the school health clinic by licensed nurses, the accuracy of which has been overseen and maintained by the general physician at the school clinic. Age was calculated as the number of completed years. All students attending this school were of Indian origin and were living as Indian expatriates in the UAE. These measurements were taken from the latest health checkup conducted in November of 2023.

Standing height was gauged using sturdy stadiometers, with students maintaining an erect posture and barefooted. Weight was assessed using precise balance scales, while students were attired in school uniforms and without footwear. Both evaluations were performed by certified and registered school nurses, endorsed by the Ministry of Health. Body mass index (BMI) was calculated using the standard formula: weight (kg)/height (m)2. Gender-specific USA growth charts were used to identify overweight (BMI ≥ 85th percentile and < 95 percentile), obese (BMI ≥ 95th percentile and < 99 percentile), morbidly or extremely obese(BMI is ≥ 99th percentile) children [14]. In addition, the World Health Organization (WHO) and International Obesity Task Force (IOTF) reference standards were also used to identify overweight, obese, and extremely obese children [15-16]. All BMI calculations were done using a website (http:// cmhsweb.uaeu.ac.ae/childbmicalculator) developed by the Ministry of Health, UAE [9].

Statistical analysis

The data was analyzed using SPSS (IBM Corp. Released 2019. IBM SPSS Statistics for Windows, Version 26.0. Armonk, NY: IBM Corp). Appropriate univariate and bivariate statistical analysis was carried out using the Student’s t-test for the continuous variable (age) and the two-tailed Fisher exact test or chi-square test for categorical variables. To measure the linear dependence between two random variables Pearson’s correlation coefficient was used. All means are expressed as mean ± standard deviation and proportion in percentages. The critical levels of significance of the results were considered at 0.05 levels i.e. P < 0.05 was considered significant. BMI categories for studied children are based on sex- and age-specific BMI percentiles using the CDC, WHO, and IOTF growth charts for children and teens aged two to 19. 

## Results

A total of 3,698 students of Gems Modern Academy, from kindergarten to 12th grade, participated in this study. The mean age of the students was 11 years, with a range of four to 18 years. Of all students, 1,727 (46.7%) were girls and 1,971 (53.3%) were boys. Further, these cohorts of students were divided into two groups, those aged 10 years or less and those older than 10 years. Out of 1,727 girls, 594 (34.4%) were aged 10 years or less, and 1,133 (65.6%) were aged more than 10 years. Similarly, out of a total of 1,971 boys, 668 (33.9%) were aged 10 years or less while 1,303 (66.1%) boys were older than 10 years. This demographic distribution of our study sample has been summarized in Table 1.

**Table 1:** Demographic profile of the study population

Gender	≤ 10 years age	> 10 years age	Total
Girls	594 (16.06%)	1,133 (30.64%)	1,727 (46.7%)
Boys	668 (18.06%)	1,303 (35.24%)	1,971 (53.3%)
Total	1,262 (34.1%)	2,436 (65.9%)	3,698 (100%)

As per CDC guidelines, obesity in children and adolescents is defined as BMI at or above the 95th percentile of sex-specific BMI-for-age, overweight is defined as between the 85th and 95th percentiles, and healthy weight is defined as between the 5th and 85th percentiles [17]. Severe obesity is defined as a gender-specific BMI greater than or equal to 120% of the 95th percentile which approximates the empirical 99th percentile at most ages [18].

**Figure 1:**
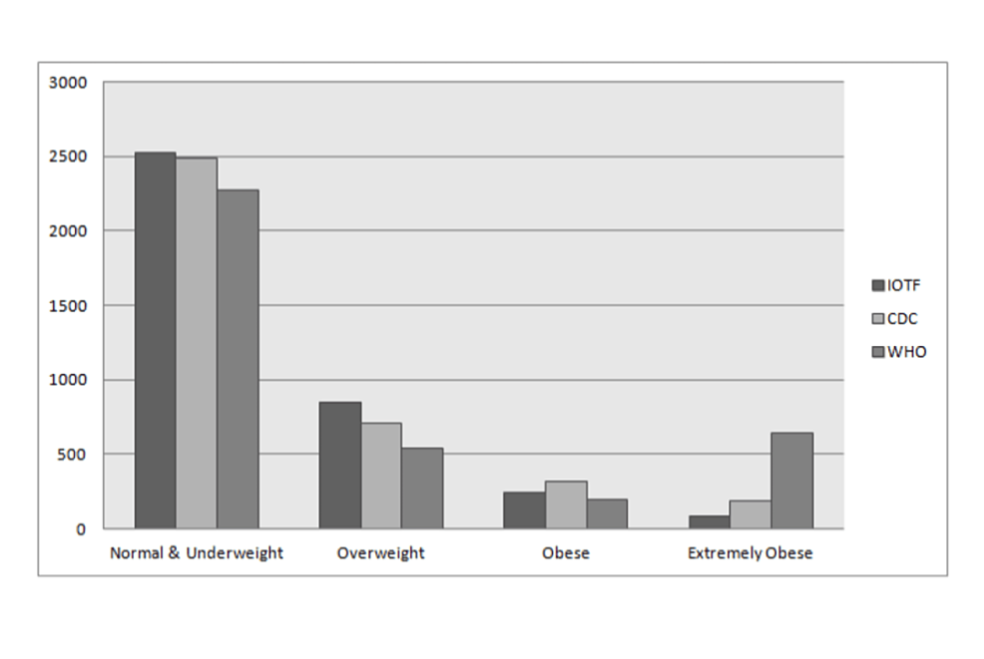
Distribution of normal & underweight, overweight, obese, and extremely obese children according to IOTF, CDC, and WHO guidelines

The prevalence of overweight, obesity, and extreme obesity based on WHO, CDC, and IOTF cutoff criteria has been shown in Tables 2, 3, 4, respectively. IOTF underestimated and WHO overestimated obesity and extreme obesity in all age groups, while CDC values were between those of IOTF and WHO (Figure 1). These results were in keeping with previously published series [9,19,20]. We have mostly used CDC reference standards for data analysis.

**Table 2:** Prevalence of overweight and obesity as per WHO criteria

Age in years	Prevalence of BMI ≥ 85th percentile						Prevalence of BMI ≥ 95th percentile					
	Girls		Boys		All (Boys + Girls)		Girls		Boys		All (Boys + Girls)	
	BMI ≥ 85 percentile	BMI < 85 percentile	BMI ≥ 85 percentile	BMI < 85 percentile	BMI ≥ 85 percentile	BMI < 85 percentile	BMI ≥ 95 percentile	BMI < 85 percentile	BMI ≥ 95 percentile	BMI < 85 percentile	BMI ≥ 95 percentile	BMI < 85 percentile
≤ 10	190 (32%)	404 (68%)	221 (33.1%)	447 (66.9%)	411 (32.6%)	851 (67.4%)	106 (17.9%)	404 (68%)	159 (23.8%)	447 (66.9%)	265 (20.7%)	851 (67.4%)
> 10	408 (36%)	725 (64%)	567 (43.5%)	736 (56.5%)	975 (40%)	1461 (60%)	229 (20.2%)	725 (64%)	352 (27%)	736 (56.5%)	581 (23.9%)	1461 (60%)
Total	598 (34.6%)	1,129 (65.4%)	788 (40%)	1,183 (60%)	1,386 (37.5%)	2,312 (62.5%)	335 (19.4%)	1,129 (65.4%)	511 (25.9%)	1,183 (60%)	846 (22.9%)	2,312 (62.5%)
	p = 0.09501		p = 0.00000746		p = 0.0001293		p = 0.1625		p = 0.008574		p = 0.01921	

**Table 3:** Prevalence of overweight and obesity as per CDC criteria

Age in years	Prevalence of BMI ≥ 85th percentile						Prevalence of BMI ≥ 95th percentile					
	Girls		Boys		All (Boys + Girls)		Girls		Boys		All (Boys + Girls)	
	BMI ≥ 85 percentile	BMI < 85 percentile	BMI ≥ 85 percentile	BMI < 85 percentile	BMI ≥ 85 percentile	BMI < 85 percentile	BMI ≥ 95 percentile	BMI < 85 percentile	BMI ≥ 95 percentile	BMI < 85 percentile	BMI ≥ 95 percentile	BMI < 85 percentile
≤ 10	176 (29.6%)	418 (70.4%)	202 (30.2%)	466 (69.8%)	378 (30%)	884 (70%)	69 (11.6%)	418 (70.4%)	106 (15.9%)	466 (69.8%)	175 (13.9%)	884 (70%)
> 10	363 (32%)	770 (68%)	470 (36%)	833 (64%)	833 (34.2%)	1,603 (65.8%)	131 (11.6%)	770 (68%)	200 (15.4%)	833 (64%)	331 (13.6%)	1,603 (65.8%)
Total	539 (31.2%)	1,188 (68.8%)	672 (34.1%)	1,299 (65.9%)	1,211 (32.8%)	2,487 (67.2%)	200 (11.6%)	1,188 (68.8%)	306 (15.5%)	1,299 (65.9%)	506 (13.7%)	2,487 (67.2%)
	p = 0.3065		p = 0.0097		p = 0.009139		p = 0.8510		p = 0.6853		p = 0.6806	

**Table 4:** Prevalence of overweight and obesity as per IOTF criteria

Age in years	Prevalence of BMI ≥ 85th percentile						Prevalence of BMI ≥ 95th percentile					
	Girls		Boys		All (Boys+ Girls)		Girls		Boys		All (Boys+ Girls)	
	BMI ≥ 85 percentile	BMI < 85 percentile	BMI ≥ 85 percentile	BMI < 85 percentile	BMI ≥ 85 percentile	BMI < 85 percentile	BMI ≥ 95 percentile	BMI < 85 percentile	BMI ≥ 95 percentile	BMI < 85 percentile	BMI ≥ 95 percentile	BMI < 85 percentile
≤ 10	166 (27.9%)	428 (72.1%)	172 (25.8%)	496 (74.2%)	338 (26.8%)	924 (73.2%)	47 (7.9%)	428 (72.1%)	56 (8.4%)	496 (74.2%)	103 (8.2%)	924 (73.2%)
> 10	376 (33.2%)	757 (66.8%)	461 (35.4%)	842 (64.6%)	837 (34.4%)	1,599 (65.6%)	94 (8.3%)	757 (66.8%)	126 (9.7%)	842 (64.6%)	220 (9.0%)	1,599 (65.6%)
Total	542 (31.4%)	1,185 (68.6%)	633 (32.1%)	1,338 (67.9%)	1,175 (31.8%)	2,523 (68.2%)	141 (8.2%)	1,185 (68.6%)	182 (9.2%)	1,338 (67.9%)	323 (8.7%)	2,523 (68.2%)
	p = 0.02581		p = 0.00001459		p = 0.000002706		p = 0.5144		p = 0.09726		p = 0.09529	

BMI for age ≥ 85th percentile (overweight, obesity, and extreme obesity)

BMI ≥ 85th percentile for their age and gender includes overweight, obese, and extremely obese children. As per CDC guidelines, a total of 1,211 children (32.74%) were overweight, obese, or extremely obese. About 1,386 (37.48 %) and 1,175 (31.77%) children had a BMI > 85th percentile by WHO and IOTF criteria, respectively. Based on CDC guidelines, of 1,727 girls, 539 (31.2%) had BMI > 85th percentile while 34.1% of boys (672/1,971) had BMI > 85 percentile.

Prevalence of > 85 percentile BMI in children over 10 years is significantly more than those aged 10 years or less (p = 0.009139 by CDC, p = 0.0001293 by WHO, and p = 0.000002706 by IOTF). Similarly, the prevalence of > 85 percentile BMI in boys aged more than 10 years was significantly more than that in boys aged 10 years or less (p = 0.0097 by CDC, p = 0.00000746 by WHO, and p = 0.00001459 by IOTF). However, in the case of girls, though the prevalence of > 85 percentile BMI was higher in girls aged > 10 years than the prevalence seen in girls aged ≤ 10 years this difference was statistically significant only with IOTF cut-offs (p = 0.02581), and not with CDC (p = 0.3065) or WHO criteria (p = 0.09501). These findings suggest that children particularly boys aged more than 10 years are at higher risk of being overweight, obese, and extremely obese.

BMI for age ≥ 95th percentile (obesity, and extreme obesity)

Children whose BMI is ≥ 95th percentile for their gender and age are classified as either obese (≥ 95 and < 99 percentile) or extremely obese (≥ 99 percentile). In our study cohort, the number of children having BMI ≥ 95th percentile was 506 (13.68%) by CDC criteria, 846 (22.88%) by WHO criteria, and 323 (8.73%) by IOTF reference standards.

Based on CDC cut-offs, 200 (11.58%) girls and 306 (15.53%) boys had BMI ≥ 95th percentile. Prevalence of ≥ 95 percentile BMI in children belonging to the > 10 years age group was 13.6 % (331/2436) and in children aged 10 years or less, the prevalence was 13.8% (175/1,262). The difference was not statistically significant (p = 0.6806). 15.3% (200/1,303) of boys that were older than ten years and 15.8% (106/668) of boys ≤ 10 years were obese or extremely obese, and the difference was not statistically significant (p = 0.6853). A similar trend was seen in girls. Rates of obesity and extreme obesity were 11.5% (131/1133)and 11.6 % (69/594) in girls older than ten years and those aged ≤ 10 years respectively, however, this difference was not statistically significant (p = 0.8510).

BMI for age ≥ 99th percentile (severe obesity)

In our study cohort of 3698 students, using CDC criteria, 191 students (5.1%) were extremely obese which included 68 girls (3.9%) and 123 boys (6.2%). Extreme obesity was more common in boys than girls however the difference was not significant statistically. The prevalence of extreme obesity in girls older than 10 years and in those aged ≤ 10 years was 3.6% and 4.5% respectively. Similarly, the prevalence of extreme obesity in boys older than 10 years and in those aged ≤ 10 years was 6.1 % and 6.4% respectively. Hence, extreme obesity is more prevalent in children who are ≤ 10 years old in comparison to older children; however, the difference was not significant statistically.

Correlation of BMI percentile by CDC criteria with age and gender

We found that there is a significant positive correlation (Pearson correlation coefficient 0.112) of BMI percentile with age and as age increases, there are significant chances of an increase in the BMI percentile. A similar positive correlation (Pearson correlation coefficient 0.164) of age with BMI percentile was seen in children ≤ 10 years. In other words, as the age increases, children, particularly those ≤ 10 years are more likely to have higher BMI percentiles. But the same trend was not observed in children aged > 10 years. For children older than 10 years, a negative (Pearson correlation coefficient -0.036) but a weak correlation of CDC percentile with age was observed. In simple words, in children older than 10 years, as the age increases, the BMI percentile decreases. However, this correlation was statistically not significant (p > 0.05).

**Figure 2:**
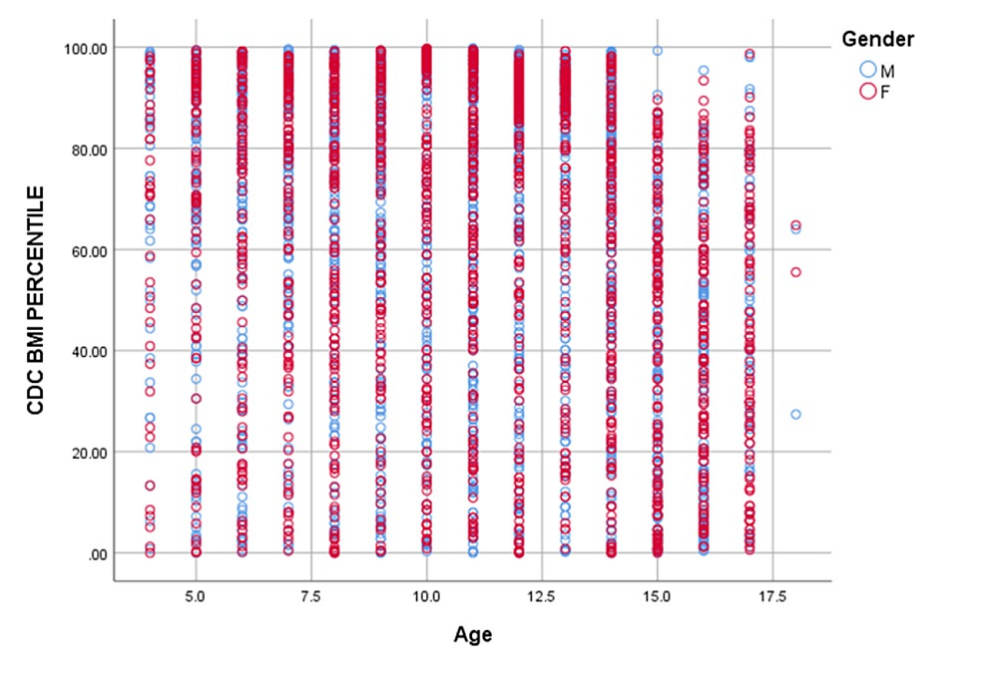
Scatter diagram depicting the distribution of CDC BMI percentile with the age and gender of students

Further, we tried to analyze the correlation of BMI percentile with respect to the gender of children (Figure 2). There is a significant strong positive correlation of BMI percentile with age in boys as well as girls aged ≤ 10 years, however, this correlation was stronger (Pearson correlation coefficient 0.227) in boys compared with girls (Pearson correlation coefficient 0.092) of the same age group. We may infer that in children aged ≤ 10 years, as the age increases, their chances of having a higher BMI percentile also increase and this is particularly prominent in boys. In children > 10 years, there is a weak negative correlation of BMI percentile with age in boys (Pearson correlation coefficient -0.027) as well as girls (Pearson correlation coefficient -0.047); however, this negative correlation is slightly more in girls but statistically both of these correlations were insignificant. To simplify, in children older than 10 years, as their age increases, their BMI percentile decreases, more prominently in girls, however, this trend is not statistically significant.

## Discussion

**Figure 3:**
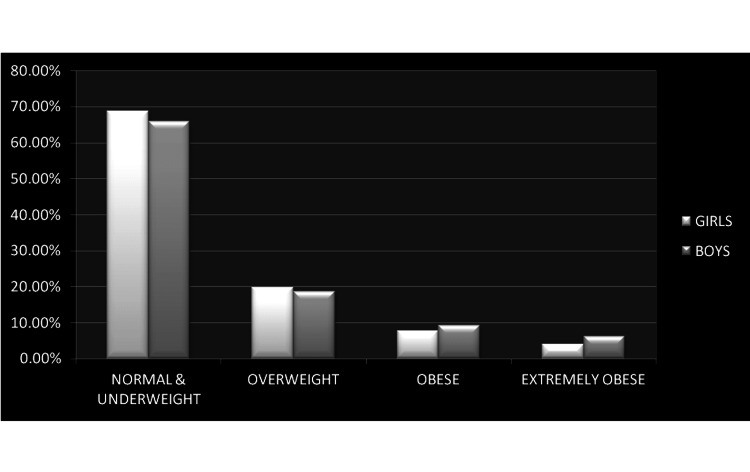
Distribution of normal & underweight, overweight, obese, and extremely obese children among girls and boys.

Though approximately one-third of the UAE population consists of Indian expatriates, to the best of our knowledge, this research is the first study aimed at estimating the burden of overweight, obesity, and severe obesity in school-aged Indian expatriates in the UAE. The interesting findings that emerge from this study include: (1) the children particularly boys > 10 years are at higher risk of being overweight, obese, and extremely obese, in comparison to the younger children; (2) in all age groups, in comparison to the girls, boys have a higher prevalence of obesity and severe obesity however the differences between two genders are not statistically significant (Figure 3); (3) in children ≤ 10 years, as the age increases, they tend to have higher BMI percentile and this is particularly prominent in boys; (4) in children > 10 years, as their age increases their BMI percentile decreases, more prominently in girls, however, this trend is not statistically significant. The trend of decreasing BMI percentile with increasing age in adolescent children, particularly girls, may be attributed to the fact that adolescent and teenage girls are often subjected to superficial standards of beauty, while this holds for both men and women, body dysmorphia and self-esteem issues can often be seen more prominently within girls [21]. Women are also more likely to take part in body weigh-ins, vomiting, dieting, avoidance, fasting, etc. [22], which could be one reasonable explanation for our findings.

Comparison of the prevalence of childhood obesity between Emiratis and Indian expatriates living in the UAE 

Many studies have been conducted to see the prevalence of obesity in school children in the UAE [9-12]. The largest such study was published by AlBlooshi et al. in 2016 in which authors investigated data of 44,942 students from government schools in Ras Al-Khaimah [9]. They collected data of 15,532 children (four to 12 years) in 2013-2014, and 29,410 children (3-18 y) in 2014-2015. This study included mostly Emirati children and concluded that using the CDC interpretation of BMI the prevalence of BMI ≥ 85th percentile was highest (41.2%) in the age range of 11-14 years. Another study published by Abduelkarem et al. in 2020 comprised data from 678 school children of six to 11 years in Sharjah and found that 28.2% of children were overweight or obese [10]. A study by Al Junaibi et al. was published in 2012 in which authors collected data from 1,541 school children aged six to 19 years from 246 schools in Abu Dhabi and found that 14.7% were overweight and 18.9% were obese [11]. A study by Baniissa et al. included adolescent children of the age range 13-19 years; 498 from public schools and 434 from private schools and they concluded that 34.7% of children had a BMI of 85 percentile or more [12].

**Figure 4:**
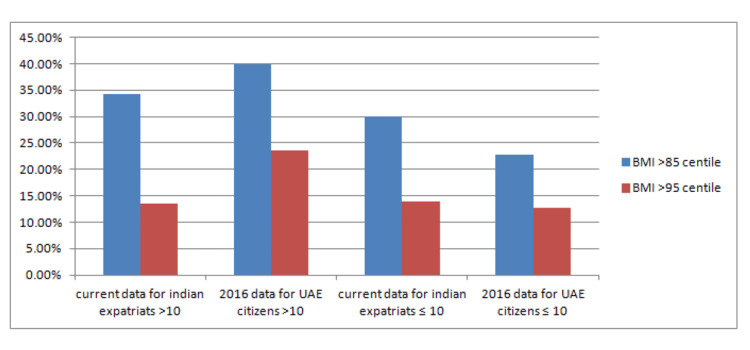
Comparison of prevalence of overweight and obesity between Indian expatriate and Emirati children

If we compare the results of our study with the paper published by AlBlooshi et al., which is the largest of the above studies and most comparable to our study population; some interesting facts do emerge (Figure 4). The prevalence of obesity in adolescent UAE citizens was estimated to be 24.3%, while in our study cohort of adolescent Indian expatriates, only 13.7% were obese. This trend was similar in both boys as well as girls. In comparison to 21.6% of adolescent girls of UAE nationality, only 11.6 % of Indian adolescent girls were obese and while 27.5% of adolescent boys of UAE nationality were found to be obese in a previous study, only 15.4 % of boys of Indian origin were obese in our cohort. Based on a previous study 39.8% of adolescent UAE citizens were found to be overweight or obese, while in our study 34.2% of Indian expatriates of the adolescent age group were either overweight or obese. Similarly, the rate of extreme obesity was 5% in Indian adolescents which was less in comparison to the 6.8% prevalence of extreme obesity in UAE citizens of adolescent age groups.

Based on this data, we can conclude that overweight and obesity are more prevalent in adolescent Emiratis in comparison to adolescent Indian expatriates. Interestingly, the same is not true for children in ≤ 10 years age group. The prevalence of overweight and obesity in ≤ 10 years Indian expatriates (30%) was higher than their Emirati counterparts (22.7%). Similarly, the previous study reported that in Emirati children of ≤ 10 years age group, 12.74% were obese and 3.58% were extremely obese. In our study cohort, rates of obesity and extreme obesity in Indian expatriates of similar age groups were 13.9% and 5.5%.

To summarize, Emirati adolescent children are more likely to be overweight and obese as compared to adolescent Indians living in the UAE however the same trend was not seen in pre-adolescent children. Rather, the prevalence of overweight and obesity was slightly higher in pre-adolescent Indian expatriates. The possible explanation may be dietary and cultural differences in Emiratis and Indians and further studies need to be done to explain and understand this.

While these inferences have been drawn by comparing our study with the largest obesity prevalence study done for UAE citizens, there are many limitations. Firstly, the study conducted on UAE citizens was based on data collected from 2013 to 2015 while our study comprises data from 2023. As the previous study concluded that the rate of obesity was increasing in Emirati children, with each year an additional 2.36% of the students becoming obese and 0.28% becoming extremely obese, it is plausible that if the rate of obesity is calculated in Emirati children in 2023, it will be higher than reported in the previous study. Secondly, our study included only 3,698 children while the previous study had data from 27078 Emirati children. Another important limitation is that our study consists of single private Indian school data while the previous study comprised data from 83 government schools.

Comparison of overweight and obesity rates among schoolchildren in India and Indian residents of the UAE

A recent meta-analysis published by Singh et al. analyzed the 21 studies published over 20 years (2003-2023) which comprised data from 186,901 children in India and estimated pooled prevalence of childhood obesity and overweight being 8.4% and 12.4% respectively [23]. Another meta-analysis published in 2016 by Ranjani et al. included data from 52 studies conducted in 16 states of India [24]. The pooled data after 2010 estimated a combined prevalence of 19.3% of childhood overweight and obesity which was a significant increase from the earlier prevalence of 16.3% reported in 2001-2005.

In our study cohort, the prevalence of overweight and obesity was 32.74% by CDC cut-off and 37.48% by WHO criteria which is much higher than that estimated in Indian children in the abovementioned two meta-analyses. Hence, we can infer that Indian children living in UAE are at higher risk of being overweight and obese compared to children living in India. The higher prevalence of obesity in high-income countries is a well-known phenomenon and the higher socio-economic status of Indians living in UAE may be a plausible explanation for the higher obesity rates in them, however, further researches are needed to explore the risk factors.

Our study had certain limitations. This was a retrospective study comprising a limited sample size from a single school. This study demonstrated the prevalence of overweight and obesity in terms of age and gender of children; however, various risk factors for childhood obesity like physical inactivity, junk food consumption, and family history were not evaluated in this research. Being cross-sectional in design, this study did not delve into the changing rate of obesity over time.

## Conclusions

Approximately one-third of school-aged Indian expatriates in the UAE are overweight, obese, or severely obese. Children particularly boys aged > 10 years are at higher risk of being overweight, obese, and extremely obese, in comparison to younger children. In children aged ≤ 10 years, as the age increases, they tend to have a higher BMI percentile.

On comparing our results with previously published research, two important trends emerge. Firstly, Emirati adolescent children are more likely to be overweight and obese as compared to adolescent Indians living in the UAE, and secondly, Indian children living in the UAE are at higher risk of being overweight and obese as compared to their counterparts in India. Further researches are required to investigate the possible risk factors for high obesity prevalence in these population groups which would guide identifying effective interventions to reduce childhood obesity.

## References

[1] (2024). Obesity and overweight. https://www.who.int/news-room/fact-sheets/detail/obesity-and-overweight.

[2] Häger A (1981). Adipose tissue cellularity in childhood in relation to the development of obesity. Br Med Bull.

[3] (2024). Prevalence of obesity. https://www.worldobesity.org/about/about-obesity/prevalence-of-obesity.

[4] (2024). Prevention and control of childhood overweight and obesity. https://www.emro.who.int/fr/health-education/prevention-and-control-of-childhood-overweight/prevention-and-control-of-childhood-overweight-and-obesity.html.

[5] (2024). Healthy weight and growth. https://www.cdc.gov/healthyweight/effects/index.html.

[6] Bibbins-Domingo K, Coxson P, Pletcher MJ, Lightwood J, Goldman L (2007). Adolescent overweight and future adult coronary heart disease. N Engl J Med.

[7] Baker JL, Olsen LW, Sørensen TI (2007). Childhood body-mass index and the risk of coronary heart disease in adulthood. N Engl J Med.

[8] (202252024). United Arab Emirates Ministry of Health & Prevention: Efforts to improve results of national indicator of prevalence of obesity. (. https://mohap.gov.ae/en/media-center/news/4/3/2022/mohap-ramps-up-efforts-to-improve-results-of-national-indicator-on-prevalence-of-obesity.

[9] AlBlooshi A, Shaban S, AlTunaiji M (2016). Increasing obesity rates in school children in United Arab Emirates. Obes Sci Pract.

[10] Abduelkarem AR, Sharif SI, Bankessli FG, Kamal SA, Kulhasan NM, Hamrouni AM (2020). Obesity and its associated risk factors among school-aged children in Sharjah, UAE. PLoS One.

[11] Al Junaibi A, Abdulle A, Sabri S, Hag-Ali M, Nagelkerke N (2013). The prevalence and potential determinants of obesity among school children and adolescents in Abu Dhabi, United Arab Emirates. Int J Obes (Lond).

[12] Baniissa W, Radwan H, Rossiter R (2020). Prevalence and determinants of overweight/obesity among school-aged adolescents in the United Arab Emirates: a cross-sectional study of private and public schools. BMJ Open.

[13] (2024). United Arab Emirates Population Statistics 2024. https://www.globalmediainsight.com/blog/uae-population-statistics/.

[14] Kuczmarski RJ, Ogden CL, Guo SS (2002). 2000 CDC Growth Charts for the United States: methods and development. Vital Health Stat 11.

[15] de Onis M, Onyango AW, Borghi E, Siyam A, Nishida C, Siekmann J (2007). Development of a WHO growth reference for school-aged children and adolescents. Bull World Health Organ.

[16] Cole TJ, Lobstein T (2012). Extended international (IOTF) body mass index cut-offs for thinness, overweight and obesity. Pediatr Obes.

[17] Ogden CL, Flegal KM (2010). Changes in terminology for childhood overweight and obesity. Natl Health Stat Report.

[18] Flegal KM, Wei R, Ogden CL, Freedman DS, Johnson CL, Curtin LR (2009). Characterizing extreme values of body mass index-for-age by using the 2000 Centers for Disease Control and Prevention growth charts. Am J Clin Nutr.

[19] Llorca-Colomer F, Murillo-Llorente MT, Legidos-García ME, Palau-Ferré A, Pérez-Bermejo M (2022). Differences in classification standards for the prevalence of overweight and obesity in children. A systematic review and meta-analysis. Clin Epidemiol.

[20] Dereń K, Wyszyńska J, Nyankovskyy S (2020). Assessment of body mass index in a pediatric population aged 7-17 from Ukraine according to various international criteria-a cross-sectional study. PLoS One.

[21] (2024). Dieting and weight worries on rise in teens. https://www.ucl.ac.uk/news/2020/nov/dieting-and-weight-worries-rise-teens.

[22] Striegel-Moore RH, Rosselli F, Perrin N, DeBar L, Wilson GT, May A, Kraemer HC (2009). Gender difference in the prevalence of eating disorder symptoms. Int J Eat Disord.

[23] Singh S, Awasthi S, Kapoor V, Mishra P (2023). Childhood obesity in India: a two-decade meta-analysis of prevalence and socioeconomic correlates. Clin Epidemiology Glob Health.

[24] Ranjani H, Mehreen TS, Pradeepa R, Anjana RM, Garg R, Anand K, Mohan V (2016). Epidemiology of childhood overweight &amp; obesity in India: a systematic review. Indian J Med Res.

